# 2-Bromoterguride–a potential atypical antipsychotic drug without metabolic effects in rats

**DOI:** 10.1007/s00213-016-4356-0

**Published:** 2016-06-17

**Authors:** Robert T. Franke, Emilia Tarland, Heidrun Fink, Heinz H. Pertz, Jan Brosda

**Affiliations:** Institute of Pharmacology and Toxicology, School of Veterinary Medicine, Freie Universität Berlin, 14195 Berlin, Germany; Institute of Pharmacy, Freie Universität Berlin, Königin-Luise-Str. 2, 14195 Berlin, Germany

**Keywords:** Dopamine D_2_ receptor partial agonist, Wet dog shakes, Conditioned avoidance response, Fos expression, Weight gain, Antipsychotic

## Abstract

**Rationale:**

Recently, we showed that 2-bromoterguride acted as a dopamine D_2_ receptor partial agonist, a serotonin 5-HT_2A_ and α_2C_-adrenergic receptor antagonist, and exhibited antidopaminergic efficacy in amphetamine-induced locomotion (AIL) in rats without inducing catalepsy.

**Objective:**

To extend our knowledge on the antipsychotic effects of 2-bromoterguride, we used convergent preclinical animal models and tests; i.e., conditioned avoidance response (CAR), predictive of antipsychotic-like effects; Fos protein expression, a molecular marker for (atypical) antipsychotic activity; wet dog shake behavior, a test for the in vivo effects of drugs acting on central 5-HT_2A_ receptors; and investigated metabolic changes as a common side effect of atypical antipsychotic drugs (APDs).

**Results:**

Acute treatment with 2-bromoterguride (0.1 and 0.3 mg/kg) decreased the CAR at 30, 90, and 270 min post-injection in rats without inducing escape failures at any time. Fos protein expression, as shown by Western blotting, was enhanced by 2-bromoterguride in the nucleus accumbens (NAc), the dorsolateral striatum (dStr), and the medial prefrontal cortex (mPFC). (±)-2,5-Dimethoxy-4-iodoamphetamine (DOI)-induced wet dog shakes in rats were reduced by 2-bromoterguride. Chronic treatment with 2-bromoterguride did not affect metabolic parameters such as body weight development and body fat composition as well as behavioral parameters such as food intake and locomotor activity.

**Conclusions:**

Our data suggest that 2-bromoterguride is a promising candidate in the treatment of schizophrenia due to its atypical antipsychotic-like activity and its inability to induce weight gain.

## Introduction

The majority of patients with schizophrenia are treated with typical or atypical antipsychotic drugs (APDs) (Leucht et al. 2009, 2013). Typical APDs such as haloperidol possess high antagonist potency at dopamine D_2_ receptors but carry substantial risk to induce extrapyramidal side effects (EPS) and increased release of prolactin (Meltzer 2013). Atypical APDs with a clozapine-like profile interact with varying affinities at D_2_, 5-HT_2A_, 5-HT_2C_, and many other monoamine receptors including α_1_- and α_2_-adrenoceptor subtypes and histamine H_1_ receptors (Kroeze et al. 2003; Leucht et al. 2009; Meltzer et al. 2012; Roth et al. 2004; Schoemaker et al. 1997). The key feature that distinguishes typical from atypical APDs is that the latter produce fewer EPS than the first (Meltzer 2013). However, atypical APDs cause considerable weight gain, which may be based, at least in part, on the antagonist properties of these drugs at histamine H_1_ and 5-HT_2C_ receptors (Kroeze et al. 2003; Roth et al. 2004).

The advent of aripiprazole, a D_2_ receptor partial agonist of moderate intrinsic activity, has generated a new treatment option in schizophrenia (DeLeon et al. 2004). D_2_ receptor partial agonists may induce a desirable stabilization of dopaminergic transmission insofar as they decrease transmission postsynaptically in the mesolimbic system and increase transmission at the mesocortical level (Lieberman 2004; Stahl 2001). Aripiprazole does not cause EPS (Leucht et al. 2013); like other atypical APDs, it shows high affinity for multiple 5-HT receptors and other central neuroreceptors (Kroeze et al. 2003).

We recently showed that 2-bromoterguride, an ergot derivative, mechanistically mimicked aripiprazole as a D_2_ receptor partial agonist (Jantschak et al. 2013). However, compared with aripiprazole, 2-bromoterguride exhibited much higher affinity for 5-HT_2A_ receptors and α_2C_-adrenoceptors and much lower affinity for histamine H_1_ receptors (Jantschak et al. 2013). Preclinical tests using rats confirmed that the multifunctional drug 2-bromoterguride had antidopaminergic activity as demonstrated by the inhibition of amphetamine-induced locomotion (AIL) and exhibited low EPS liability as shown by the failure to produce catalepsy. The in vivo observations in conjunction with its broad neuroreceptor binding profile suggest that 2-bromoterguride may be classified as an atypical APD.

The aim of the present study was to extend our knowledge on the antipsychotic effects of 2-bromoterguride. We examined whether 2-bromoterguride might inhibit the conditioned avoidance response (CAR), a widely accepted screening tool in rats with high predictive validity that has shown particular sensitivity for detection of potential antipsychotic activity (Wadenberg 2010). It is well documented that APDs alter Fos expression in nucleus accumbens (NAc), dorsolateral striatum (dStr), and medial prefrontal cortex (mPFC) (Robertson et al. 1994; Sumner et al. 2004). Therefore, we measured 2-bromoterguride-induced Fos protein expression in these brain regions with special focus on the effects in the mPFC because a Fos enhancement in this region can be used to identify atypical APDs (Robertson and Fibiger 1996). To substantiate the 5-HT_2A_ receptor antagonist potency of 2-bromoterguride in vivo, it seemed reasonable to find out whether the drug was able to reduce (±)-2,5-dimethoxy-4-iodoamphetamine (DOI)-induced wet dog shakes in rats, a test for drugs acting on central 5-HT_2A_ receptors (Schreiber et al. 1995). As mentioned above, atypical APDs can induce weight gain. Therefore, we analyzed the effects of prolonged 2-bromoterguride administration on food intake, weight gain, and body fat composition, as well as on food and water consumption. Together with the preclinical tests of our previous study, AIL and catalepsy (see Jantschak et al. 2013), we conclude that 2-bromoterguride has atypical antipsychotic properties without metabolic side effects such as weight gain.

## Materials and methods

### Animals

All experiments were performed in accordance with the guidelines of the German Animal Protection Law and approved by the Berlin State Authority (“Landesamt für Gesundheit und Soziales”). A total of 163 naïve male (acute experiments) and female (chronic experiment) Sprague-Dawley rats (Élevage Janvier, Le Genest Isle, France) at an age of 10–13 weeks were used. The animals were housed in groups of two to three in type IV open-top polycarbonate cages (Ehret, Emmendingen, Germany) with dust-free hardwood bedding (Hygienic Animal Bedding; J. Rettenmaier & Söhne, Rosenberg, Germany) under standard conditions (room temperature, 22 ± 2 °C; relative humidity 55 ± 10 %) on a 12-h light-dark schedule (lights on at 6:00 a.m.). Home cages were enriched by paper tissues and metal tubes as hiding places. Rats had free access to standard laboratory chow (ssniff, Soest, Germany) and tap water and were handled and weighed regularly. Once weekly, the home cages were cleaned and equipped with new bedding by a professional animal keeper. Animals were free of pathogens according to Federation for Laboratory Animal Science (FELASA) recommendations.

### Drugs

2-Bromoterguride (Alfarma sro, Cernosice, Czech Republic) and olanzapine (Sigma-Aldrich, Steinheim, Germany) were suspended in 15 % Cremophor® EL (Sigma-Aldrich, Steinheim, Germany). Aripiprazole (Toronto Research Chemicals, Toronto, Canada) was dissolved in *N*,*N*-dimethylformamide (30 %) blended with acetic acid (1 %). (±)-2,5-Dimethoxy-4-iodoamphetamine hydrochloride (DOI; Sigma-Aldrich, Steinheim, Germany), ketanserin tartrate, and haloperidol (Janssen Pharmaceuticals, Beerse, Belgium) were dissolved in 0.9 % saline. All drug solutions were freshly prepared before being injected intraperitoneally (i.p.; injection volume 1 ml/kg body weight).

### Conditioned avoidance response

Male rats were trained and tested in two computer-assisted, two-way active avoidance shuttle boxes (Med Associates Inc., St. Albans, USA), which were enclosed by MDF sound-attenuating cubicles and equipped with stimulus lights, tone generators, and grid floors. Each shuttle box consisted of two compartments of equal size separated by a partition with an aperture, which allowed the rats to move freely between the compartments. The positions of the animals were tracked by eight infrared photobeams. Each session (training, drug-free pre-test, drug-test) started with a 3-min habituation period, in which no stimuli presentation occurred, followed by 30 trials. Upon presentation of a white noise (76 dB) and light as conditioned stimuli (CS), rats needed to move from one compartment into the other within 10 s to avoid the unconditioned stimulus (UCS), which was presented for 20 s as an intermittent electric shock in the grid floor (0.3 mA; interval 2 s; single shock duration 0.5 s). Moving from one compartment to the other one within the first 10 s (response to CS) was recorded as avoidance, and a change as a response to the UCS was recorded as an escape. Escape failures were recorded if an animal failed to respond to the CS and USC within the trial duration of 30 s. Inter-trial intervals randomly varied between 10 and 30 s. Rats were daily trained until they met the test criterion (≥80 % avoidances) on two consecutive days. The next day, a drug-free pre-test was performed. Subsequently, the animals were injected with 2-bromoterguride (0.1 and 0.3 mg/kg), haloperidol (0.05 and 0.1 mg/kg), aripiprazole (1 and 3 mg/kg), or the respective vehicle. The CAR was evaluated 30, 90, and 270 min and 24 h post-injection as percent avoidance compared to percent avoidance of the pre-test and percent avoidance of the control group, respectively. The doses for aripiprazole and haloperidol were determined by conducting pilot studies. Each compound was tested in one treatment group. The animals of the three treatment groups were tested repeatedly in a pseudorandom order serving as their own controls (wash-out period of at least 4 days).

### Neuronal Fos expression

Male rats were transferred within their home cages to a sound-proof red lighted dark-room for 2 days. During this period, they were habituated to injections of saline. On day 3, rats were injected with 2-bromoterguride (0.1 and 0.3 mg/kg), haloperidol (0.5 mg/kg), or vehicle (15 % Cremophor® EL). The dose of haloperidol was determined by conducting a pilot study. Two hours later, animals were sacrificed by decapitation and brains rapidly removed from the skull. Brain areas were dissected on a cold plate. The following tissue samples were weighed and shock frozen in liquid nitrogen: mPFC, NAc, and dStr (Heffner et al. 1980).

Expression of Fos and β-actin in brain homogenates were studied by Western blotting using the following primary antibodies: rabbit anti-Fos (1:2000) and rabbit anti-β-actin as loading control (1:20,000; New England BioLabs, Frankfurt a. M., Germany). Bands were detected by probing with a horseradish peroxidase-conjugated goat anti-rabbit secondary antibody (1:10,000; Cell Signaling Technology, Leiden, The Netherlands). Visualization was achieved by chemiluminescence with ECL Prime Western Blotting Detection Reagent (Amersham, Braunschweig, Germany) on Amersham Hyperfilm (GE Healthcare Limited, Little Chalfont, UK). Quantification of bands was obtained by digital image analysis using National Institutes of Health Image software (http://rsb.info.nih.gov/ij/download.html).

### Antagonism of DOI-induced wet dog shakes

The experiments were conducted in two glass cuboids (29 × 22 × 39 cm) of which the grounds were coated with bedding material. Male rats were given an injection of either 2-bromoterguride (0.1 or 0.3 mg/kg), aripiprazole (3 mg/kg), ketanserin (1 mg/kg), or vehicle (15 % Cremophor® EL). After 30 min, animals were injected with DOI (2 mg/kg) and vehicle, respectively. Subsequently, the behavior of the animals was monitored and recorded (Canon HG10 HD-Camcorder; Canon, Krefeld, Germany) for 30 min. A trained observer of rat behavior determined the number of wet dog shakes.

### Body weight development, body fat composition, food intake, locomotor activity, and cataleptic behavior

Female rats were used as they exhibit a more robust weight gain following atypical APD treatment compared to males (Choi et al. 2007; Davey et al. 2012). However, in female rats, clozapine does not increase weight gain mandatorily but does increase visceral fat tissue (Cooper et al. 2008). Therefore, we used a comprehensive approach by investigating the effects of 2-bromoterguride on body fat composition and body weight development with olanzapine (instead of clozapine) as a positive control. Animals were matched into four different treatment groups based on their body weight. Rats were injected B.I.D. (first injection between 9:00 and 10:00 a.m.; second injection between 4:00 and 5:00 p.m.) with 2-bromoterguride (0.1 and 0.3 mg/kg), olanzapine (2 mg/kg), or vehicle (15 % Cremophor® EL) for 21 days. Body weight, food and water intake were measured daily prior to the first injection. Weight gain (%) was calculated in relation to body weight of day 0. The amount of food and water consumed was calculated in grams or milliliters per cage (*n* = 2) in relation to body weight. On day 22, rats were deeply anaesthetized with chloral hydrate (720 mg/kg; Sigma-Aldrich, Steinheim, Germany) and visceral (gonadal and retroperitoneal), subcutaneous (inguinal), and brown fat tissue (interscapular) were dissected and weighed by a trained experimenter. Tissue weights were calculated in relation to body weight. In order to compare the effect of acute versus prolonged 2-bromoterguride treatment on behavior, we analyzed locomotor activity in the open field (day 14; distance traveled (cm)) and cataleptic behavior (day 20; bar and grid test) as described previously (Jantschak et al. 2013).

### Data presentation and analysis

Data were analyzed and presented using SigmaPlot 11 (Systat Software, Erkrath, Germany). The following tests were used: one-way analysis of variance (ANOVA) (Fos expression, wet dog shakes, body fat composition, locomotor activity) and two-way repeated measures (RM) ANOVA (treatment × time) (CAR, body weight development, food intake) followed by Holm-Sidak *t* tests. Data of escape failures and cataleptic behavior were not normally distributed (tested with the Shapiro-Wilk method) and analyzed with non-parametric Friedman one-way RM ANOVA and Kruskal-Wallis one-way ANOVA followed by Dunn’s method, respectively. *P* values <0.05 were considered to be significant. Data are presented as mean values ± standard error of the means (SEM).

## Results

### Conditioned avoidance response

**Fig. 1:**
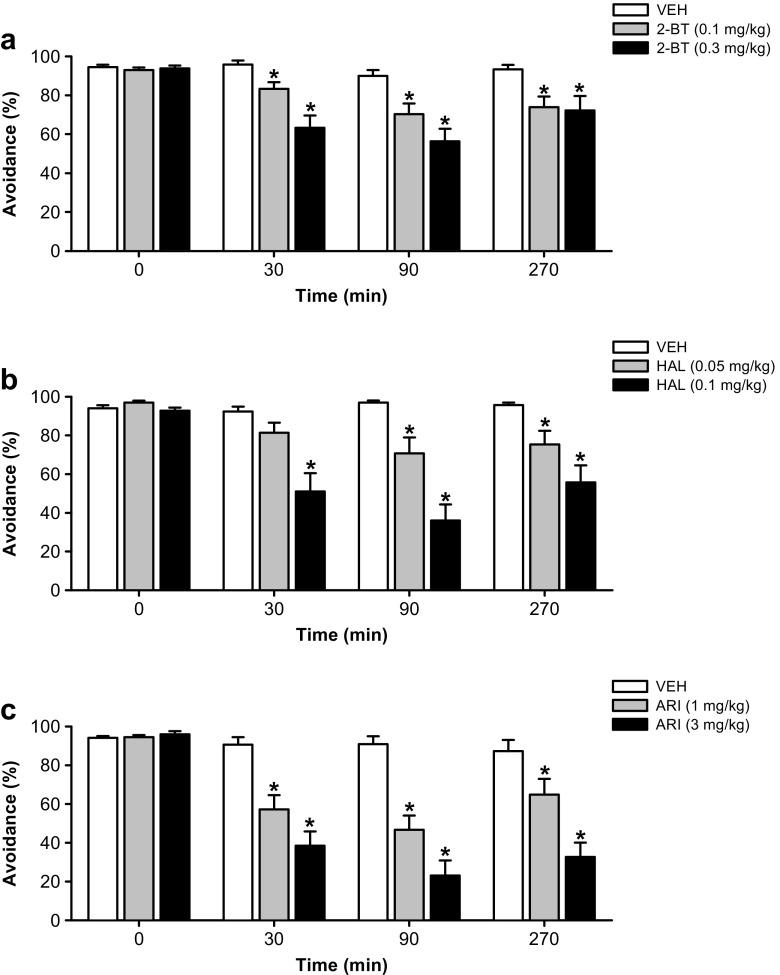
Effects of 2-bromoterguride (0.1 and 0.3 mg/kg; **a**), haloperidol (0.05 and 0.1 mg/kg; **b**), or aripiprazole (1 and 3 mg/kg; **c**) on percentage conditioned avoidance response at 30, 90, and 270 min after injection. Data are expressed as mean ± SEM of 12 (2-bromoterguride, aripiprazole) or 10 male rats (haloperidol). ^*^ *P* < 0.05 vs. vehicle. *2-BT* 2-bromoterguride, *ARI* aripiprazole, *HAL* haloperidol, *VEH* vehicle

2-Bromoterguride, haloperidol, and aripiprazole inhibited CAR in a dose-dependent manner (Fig. 1). *2*-*Bromoterguride*: significant effects for the factors treatment (*F*_(2,88)_ = 12.7, *P* < 0.001), time (*F*_(4,88)_ = 23.3, *P* < 0.001), and for the interaction of the factors (*F*_(8,88)_ = 4.7, *P* < 0.001) were observed. Both doses of 2-bromoterguride (0.1 and 0.3 mg/kg) decreased the CAR at 30, 90, and 270 min post-injection compared to pre-tests (*P* < 0.01) and controls (*P* < 0.001) without inducing escape failures at any time (Fig. 1a). *Haloperidol*: a two-way RM ANOVA showed significant effects for the factors treatment (*F*_(2,72)_ = 16.3, *P* < 0.001), time (*F*_(4,72)_ = 23.6, *P* < 0.001), and for the interaction of the factors (*F*_(8,72)_ = 9.1, *P* < 0.001). Haloperidol (0.1 mg/kg) decreased the CAR at 30 min post-injection, whereas 0.05 and 0.1 mg/kg haloperidol showed this effect at 90 and 270 min after administration compared to pre-tests (*P* < 0.001) and controls (*P* < 0.01) without inducing any escape failures (Fig. 1b). *Aripiprazole*: significant effects for the factors treatment (*F*_(2,80)_ = 38.5, *P* < 0.001), time (*F*_(4,80)_ = 49.1, *P* < 0.001), and for the interaction of the factors (*F*_(8,80)_ = 12.9, *P* < 0.001) were revealed. Both doses of aripiprazole (1 and 3 mg/kg) decreased the CAR at 30, 90, and 270 min post-injection compared to pre-tests (*P* < 0.001) and controls (*P* < 0.01; Fig. 1c). Aripiprazole (3 mg/kg) increased escape failures 30 min (escape failures–vehicle 0 ± 0, aripiprazole 3.2 ± 1.4; *χ*^2^ = 13.6, *P* < 0.01, *df* = 2) and 90 min (escape failures–vehicle 0 ± 0, aripiprazole 4.7 ± 1.7; *χ*^2^ = 7.7, *P* < 0.05, *df* = 2) after administration compared to controls. All drugs failed to affect CAR behavior after 24 h.

### Neuronal Fos induction

**Fig. 2:**
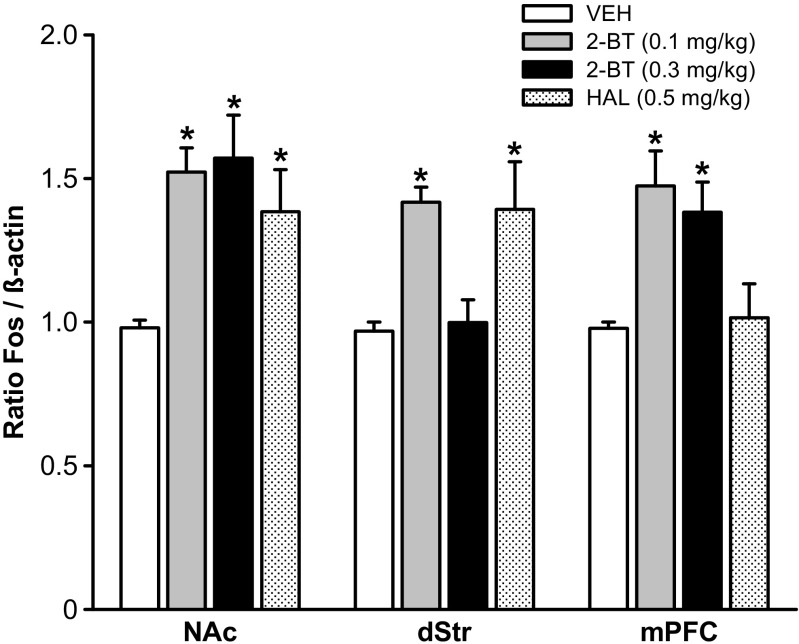
Effects of 2-bromoterguride (0.1 and 0.3 mg/kg) or haloperidol (0.5 mg/kg) on Fos protein expression in the nucleus accumbens (*NAc*), dorsal striatum (*dStr*), and medial prefrontal cortex (*mPFC*). Data are expressed as mean ± SEM of 8 male rats each treatment group. *P < 0.05 vs. vehicle. *2-BT* 2-bromoterguride, *HAL* haloperidol, *VEH* vehicle

2-Bromoterguride increased Fos levels in the striatum and mPFC, whereas haloperidol induced an enhancement of Fos expression only in the striatum (Fig. 2). One-way ANOVA revealed significant treatment effects in the mPFC (*F*_(3,28)_ = 6.8; *P* < 0.001), NAc (*F*_(3,26)_ = 5.6; *P* = 0.004), and dStr (*F*_(3,27)_ = 5.8; *P* < 0.003). 2-Bromoterguride (0.1 and 0.3 mg/kg) enhanced Fos expression both in the mPFC (*P* < 0.05) and NAc (*P* < 0.01). In the dStr, only the lower dose of 2-bromoterguride induced an increase of the Fos level (*P* < 0.05). Haloperidol increased neuronal activity in the NAc and dStr (*P* < 0.05), but not in the mPFC (Fig. 2).

### Antagonism of DOI-induced wet dog shakes

2-Bromoterguride attenuated DOI-induced wet dog shakes (Table 1). One-way ANOVA revealed significant treatment effects in wet dog shaking behavior (*F*_(5,53)_ = 10.8; *P* < 0.001). DOI (5-HT_2A/2C_ receptor agonist) induced a robust increase in wet dog shakes (*P* < 0.001). Pretreatment with ketanserin (5-HT_2A_ receptor antagonist) suppressed the effect of DOI to vehicle level (*P* < 0.001). 2-Bromoterguride (0.1 and 0.3 mg/kg) also reduced DOI-induced wet dog shakes (*P* < 0.05); however, the effect was different from vehicle (*P* < 0.05). Unlike 2-bromoterguride, the reduction of the DOI effect by aripiprazole was not significant, although it was significant versus vehicle (*P* < 0.05; Table 1).

**Table 1:** Effects of ketanserin (1 mg/kg), 2-bromoterguride (0.1 and 0.3 mg/kg), or aripiprazole (3 mg/kg) on the number of DOI (2 mg/kg)-induced wet dog shakes

Treatment	Number of wet dog shakes
VEH + VEH	0.60 ± 0.43
VEH + DOI (2 mg/kg)	13.00 ± 2.00*
KET (1 mg/kg) + DOI (2 mg/kg)	0.38 ± 0.18^#^
2-BT (0.1 mg/kg) + DOI (2 mg/kg)	7.18 ± 1.35^#^
2-BT (0.3 mg/kg) + DOI (2 mg/kg)	6.64 ± 1.88^#^
ARI (3 mg/kg) + DOI (2 mg/kg)	8.38 ± 1.13*

Data are expressed as mean ± SEM of 11 (2-bromoterguride, DOI), 10 (vehicle), or 8 male rats (aripiprazole, ketanserin)

*2*-*BT* 2-bromoterguride, *ARI* aripiprazole, *KET* ketanserin, *VEH* vehicle

*P < 0.05 vs. vehicle; ^#^ *P* < 0.05 vs. DOI

### Body weight development, body fat composition, food intake, locomotor activity, and cataleptic behavior

**Fig. 3:**
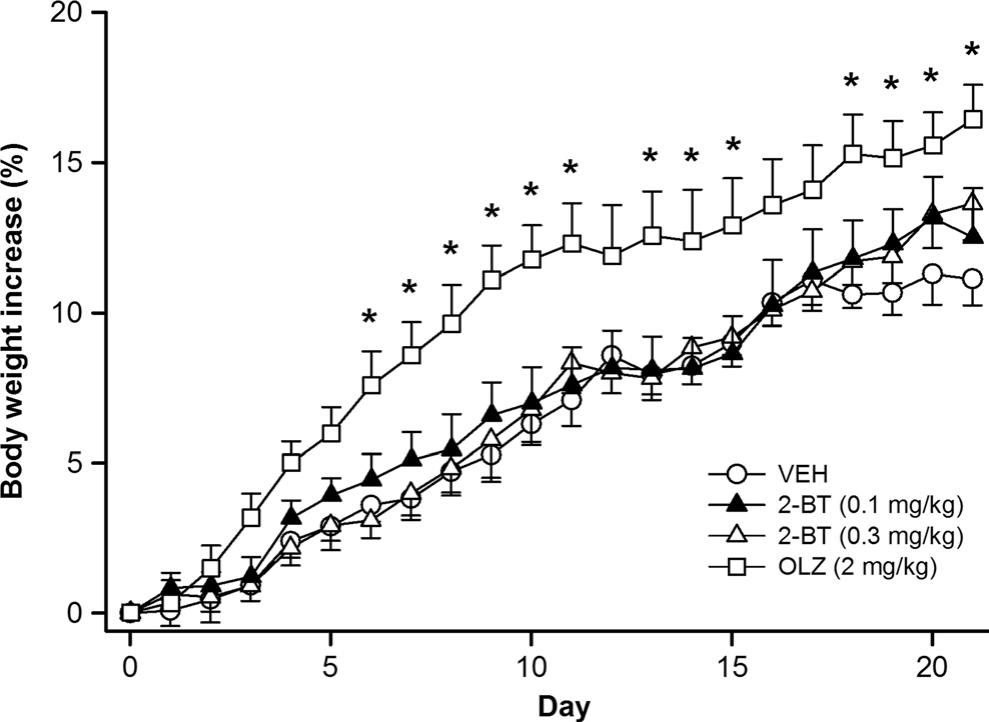
Effects of 2-bromoterguride (0.1 and 0.3 mg/kg) or olanzapine (2 mg/kg) on percentage weight gain in female rats treated for 21 days B.I.D. Data are expressed as mean ± SEM of 9 (2-bromoterguride, vehicle) or 8 rats (olanzapine). ^*^ *P* < 0.05 vs. vehicle. *2-BT* 2-bromoterguride, *OLZ* olanzapine, *VEH* vehicle

**Fig. 4:**
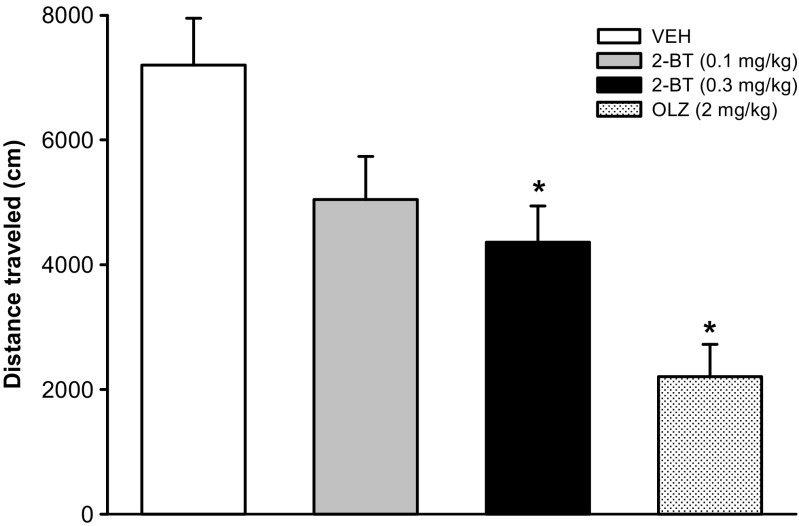
Effects of 2-bromoterguride (0.1 and 0.3 mg/kg) or olanzapine (2 mg/kg) on spontaneous locomotion plotted as total distance traveled in 30 min. Data are expressed as mean ± SEM of 9 (2-bromoterguride, vehicle) or 8 rats (olanzapine). ^*^ *P* < 0.05 vs. vehicle. *2-BT* 2-bromoterguride, *OLZ* olanzapine, *VEH* vehicle

2-Bromoterguride did not affect body weight, body fat composition, or food intake. *Body weight development*: main effects for the factors treatment (*F*_(3,651)_ = 4.8, *P* = 0.008), time (*F*_(21,651)_ = 196.5, *P* < 0.001), and for the interaction of the factors (*F*_(63,651)_ = 1.8, *P* < 0.001) were observed. Olanzapine (2 mg/kg) increased body weight (days 6–11, 13–15, 18–21; *P* < 0.05), whereas 2-bromoterguride (0.1 and 0.3 mg/kg) did not (Fig. 3). *Body fat composition*: olanzapine (2 mg/kg) increased the amount of visceral (*F*_(3,31)_ = 6.3; *P* = 0.002) and subcutaneous (*F*_(3,31)_ = 11.1; *P* < 0.001) fat tissue compared to controls (*P* < 0.01). 2-Bromoterguride did not affect fat tissues (Table 2). *Food intake*: statistical analysis revealed main effects for the factors treatment (*F*_(3,651)_ = 5.8, *P* = 0.003), time (*F*_(21,651)_ = 14.1, *P* < 0.001), and for the interaction of the factors (*F*_(63,651)_ = 3.4, *P* < 0.001). Animals treated with olanzapine (2 mg/kg) had a higher food intake (days 2–10; *P* < 0.05), whereas rats treated with 2-bromoterguride showed no difference compared to controls (*P* > 0.05; data not shown). *Water intake*: main effects for the factors treatment (*F*_(3,651)_ = 49.7, *P* < 0.001), time (*F*_(21,651)_ = 12.9, *P* < 0.001), and for the interaction of the factors (*F*_(63,651)_ = 2.9, *P* < 0.001) were observed. 2-Bromoterguride decreased (days 1–21; *P* < 0.001), whereas olanzapine (2 mg/kg) increased water consumption (days 1–3, 5–6, 9–21; *P* < 0.05) (data not shown). *Locomotor activity and cataleptic behavior*: locomotor activity was affected (*F*_(3,31)_ = 9.5; *P* < 0.001). Rats treated with 2-bromoterguride (0.3 mg/kg) and olanzapine traveled a shorter distance compared to controls (*P* < 0.05) (Fig. 4). No treatment effects were detected in the bar and grid test.

**Table 2:** Effects of 2-bromoterguride (0.1 and 0.3 mg/kg) or olanzapine (2 mg/kg) on visceral, subcutaneous, and brown fat tissue in female rats treated for 21 days B.I.D.

Treatment	Visceral fat tissue	Subcutaneous fat tissue	Brown fat tissue
VEH	2.24 ± 0.20	1.09 ± 0.07	0.08 ± 0.008
2-BT (0.1 mg/kg)	2.14 ± 0.17	1.10 ± 0.05	0.09 ± 0.009
2-BT (0.3 mg/kg)	2.02 ± 0.11	1.10 ± 0.04	0.06 ± 0.005
OLZ (2 mg/kg)	2.93 ± 0.14*	1.61 ± 0.13*	0.08 ± 0.004

Tissue weights were calculated in relation to body weight (g/100 g bw). Data are expressed as mean ± SEM of 9 (2-bromoterguride, vehicle) or 8 rats (olanzapine)

*2*-*BT* 2-bromoterguride, *OLZ* olanzapine, *VEH* vehicle

^*^ *P* < 0.05 vs. vehicle

## Discussion

The present study further demonstrates that 2-bromoterguride, a drug with partial agonist effects at D_2_ receptors, high affinity for 5-HT_2A_ and α_2C_-adrenergic but low affinity for histamine H_1_ receptors (Jantschak et al. 2013), has antipsychotic properties without inducing weight gain. In our previous study, we investigated the antidopaminergic efficacy of 2-bromoterguride using AIL, and EPS liability using catalepsy tests (Jantschak et al. 2013). The CAR provides a further highly predictive and reliable screening tool to test potential drugs exhibiting antipsychotic-like properties (Wadenberg 2010). It has been shown that well-established APDs, within a certain dose range, effectively suppress CAR without inducing escape failures. The incidence of escape failures at a given dose indicates that this dose produces non-specific behavioral effects such as sedation (Wadenberg 2010). Effective suppression of CAR with typical and atypical APDs in rats can be achieved by a striatal D_2_ receptor occupancy (D_2_RO) of 65–80 % (for aripiprazole a D_2_RO of >85 % was required to inhibit CAR). These D_2_ROs reflect the range in which schizophrenic patients respond to APDs (Natesan et al. 2006; Wadenberg et al. 2001; Wadenberg 2010). The present study shows that 2-bromoterguride, haloperidol, and aripiprazole produced a suppression of CAR in a comparable and dose-dependent manner. Only the high dose of aripiprazole induced more escape failures than vehicle. This may be related to the relatively high affinity of aripiprazole for H_1_ receptors; haloperidol and 2-bromoterguride exhibit low affinities for these receptors (Jantschak et al. 2013; Kroeze et al. 2003). It should be noted that other receptors than the D_2_ receptor may mediate or contribute to the suppression of CAR by 2-bromoterguride. For example, a blockade of 5-HT_2A_ receptors may be involved in the disruptive effect on CAR (Wadenberg et al. 1998). Consistent with this hypothesis, the acute effect of clozapine on avoidance responding was reversed by DOI (Li et al. 2012). Although DOI is non-selective for 5-HT_2A_ versus 5-HT_2C_ receptors, the former receptor population can be favored to be involved in drug-induced disruption on CAR (Halberstadt et al. 2009; Li et al. 2010, 2012; Schreiber et al. 1995; Sipes and Geyer 1995; Smith et al. 2003). In contrast to the effect of clozapine, the inability of DOI to reverse CAR disruption by olanzapine was explained by a predominant involvement of D_2_ receptors in this response (Li et al. 2012). This is because olanzapine has a much higher D_2_ receptor affinity than clozapine, whereas both drugs display similar affinity for 5-HT_2A_ receptors (Kroeze et al. 2003). 2-Bromoterguride exhibits high affinity for both D_2_ and 5-HT_2A_ receptors; therefore, it is difficult to decide which receptor is predominantly responsible for the inhibition of CAR by this drug. α_2_-Adrenoceptor blockade may also contribute to the effect of 2-bromoterguride on CAR. Pretreatment with idazoxan (α_2_-adrenoceptor antagonist) enhanced the antipsychotic effects of typical and atypical APDs, as, for example, demonstrated in the CAR test (Marcus et al. 2010). Because 2-bromoterguride exhibited high affinity for D_2_, 5-HT_2A_, and α_2C_-adrenoceptors, all of these properties may be associated with its ability to suppress CAR, thus highlighting the antipsychotic-like potential of this drug.

APDs induce the expression of the immediate-early gene product Fos in a distinct pattern of brain regions, which is suggested as an indicator of antipsychotic efficacy (Dragunow et al. 1990; Miller 1990; Robertson et al. 1994; Natesan et al. 2011). In addition, expression of Fos in the mPFC can be used to identify atypical APDs (Robertson and Fibiger 1996). For example, acute administration of haloperidol increased Fos expression in the dStr and the NAc but not in the mPFC (Young et al. 1999; this study). In contrast, clozapine and other atypical APDs enhanced Fos protein in the NAc and mPFC but not in the dStr (Deutch et al. 1992; Dilts et al. 1993; Nguyen et al. 1992; Robertson et al. 1994; Robertson and Fibiger 1996). In this context, it was of special interest to find out whether 2-bromoterguride, a low-efficacy D_2_ receptor partial agonist, might enhance Fos in different brain regions. As suggested by Natesan et al. (2011), a criterion for therapeutic success of novel APDs may be their potential to exhibit low intrinsic activity coupled with sufficient D_2_ receptor functional antagonism. Among several preclinical tests, Natesan et al. (2011) favored Fos expression in the NAc as a marker of overall functional antagonism and thus high predictive validity. 2-Bromoterguride increased Fos expression in the NAc, the dStr, and the mPFC as well. This means that 2-bromoterguride mimics haloperidol as an enhancer of Fos in the NAc and the dStr, and clozapine in the NAc and the mPFC. Positive symptoms of schizophrenia are related to an excess of dopamine in the NAc, whereas a lack of dopamine in the dorsolateral PFC is essential for negative symptoms and cognitive deficits (Abi-Dargham 2004). The increase of Fos in the NAc and the mPFC in rats by 2-bromoterguride suggests that this drug may possess the beneficial antipsychotic properties of both haloperidol and clozapine and thus may contribute to improve treatment outcome. Admittedly, this suggestion is rather speculative and further studies are needed to substantiate the findings of our study. Anyway, particularly the increase of Fos in the mPFC by 2-bromoterguride indicates that this drug may have the antipsychotic properties of an atypical APD with respect to actions on negative symptoms and cognitive deficits in schizophrenia (Robertson et al. 1994; Robertson and Fibiger 1996).

Wet dog shakes are behavioral effects in rodents which are amenable to inhibition by a large number of 5-HT receptor antagonists including APDs (Porsolt et al. 2010). The wet dog shake response is induced by selective activation of central 5-HT_2A_ receptors (Schreiber et al. 1995). 5-HT_2A_ receptor inverse agonist/antagonist activity is a characteristic feature of atypical APDs (Meltzer et al. 2012). The high in vitro affinity for 5-HT_2A_ receptors is in line with the effectiveness of 2-bromoterguride to reduce DOI-induced wet dog shakes in rats. In contrast to the reduction of DOI-induced wet dog shakes by 2-bromoterguride (0.1 and 0.3 mg/kg), the decrease by aripiprazole (3 mg/kg) was not significant. It should be emphasized that aripiprazole attenuated DOI-induced wet dog shakes when high doses were used (10–30 mg/kg; Kohnomi et al. 2008), reflecting the low affinity of aripiprazole for 5-HT_2A_ receptors. Indeed, 5-HT_2A_ receptor affinity of aripiprazole was 50-fold lower than that of 2-bromoterguride (Jantschak et al. 2013). The difference between ketanserin and 2-bromoterguride as inhibitors of DOI-induced wet dog shakes needs a comprehensive consideration. Although both compounds exhibited similar 5-HT_2A_ receptor affinity, ketanserin reduced the DOI response to vehicle level, whereas 2-bromoterguride did not. A possible explanation for this discrepancy may be that ketanserin is a competitive 5-HT_2A_ receptor antagonist in contrast to 2-bromoterguride which behaves as an insurmountable antagonist (Jantschak et al. 2013). Moreover, terguride, the parent drug of 2-bromoterguride, was a partial agonist at 5-HT_2A_ receptors in a GTPγS binding assay (Newman-Tancredi et al. 2002). It cannot be excluded that 2-bromoterguride is acting as a partial agonist at 5-HT_2A_ receptors. Furthermore, 2-bromoterguride displayed subnanomolar affinity for α_2C_-adrenoceptors (Jantschak et al. 2013). Interestingly, α_2_-adrenoceptor antagonists reversed the suppression of head-twitch behavior by 5-HT_2A_ receptor antagonists (Matsumoto et al. 1997). In addition, 2-bromoterguride may modulate AMPA/kainate receptors. Indeed, blockade of AMPA/kainate receptors attenuated DOI-induced wet dog shakes (Gorzalka et al. 2005).

Clinically significant weight gain is a serious side effect of atypical APDs (Correll et al. 2011 for review; Parsons et al. 2009) and characterized by a substantial increase in food consumption and visceral and subcutaneous fat deposition (Zhang et al. 2004). A recent meta-analysis showed that haloperidol, ziprasidone, and lurasidone were the only APDs without affecting body weight in adults (Leucht et al. 2013). Since olanzapine induced more weight gain than any other APD in preclinical and clinical studies (Allison et al. 1999; Davey et al. 2012; Leucht et al. 2013), we used this drug as a positive control. Olanzapine-induced weight gain, food consumption as well as visceral and subcutaneous fat tissue weights in rats are consistent with observations of other groups (Choi et al. 2007; Davey et al. 2012; Mann et al. 2013). The mechanism by which olanzapine and other APDs induce weight gain is likely multifactorial and not completely understood. It has been suggested that 5-HT_2C_ and H_1_ receptors, for which olanzapine and clozapine display high affinity, are strong candidate receptors for affecting appetite and thereby weight gain (Deng et al. 2010; Kirk et al. 2009; Kroeze et al. 2003). In addition, association of a polymorphism of the 5-HT_2C_ receptor gene with APD-induced weight gain highlights the role of this receptor in this metabolic side effect (Reynolds et al. 2002). However, mechanisms other than those mentioned above, e.g., D_2_ receptor gene polymorphism, may also be associated with APD-induced weight gain (Müller et al. 2012).

In contrast to the effect of olanzapine, chronic treatment of rats with 2-bromoterguride failed to elicit an effect on body weight and body fat proportion. The low affinity of 2-bromoterguride for H_1_ receptors (pA_2_ 6.0; Jantschak et al. 2013) may be responsible for the non-occurrence of this side effect in the treatment with this drug. Unfortunately, there are no data available with regard to the affinity of 2-bromoterguride for 5-HT_2C_ receptors. However, terguride, the parent drug of 2-bromoterguride, exhibited low affinity for this receptor (p*K*_B_ 6.1; Newman-Tancredi et al. 2002). The absence of any effect of 2-bromoterguride on body weight, food consumption, and visceral and subcutaneous fat tissue weights suggests that 5-HT_2C_ receptors have no functional relevance in the side effect profile of this drug. Further, chronic treatment with 2-bromoterguride decreased locomotor activity but failed to induce catalepsy. This is consistent with the effect of acute 2-bromoterguride treatment (Jantschak et al. 2013) and other D_2_ receptor partial agonists (Natesan et al. 2011; Nordquist et al. 2008; Svensson et al. 1991). The sedative effect of 2-bromoterguride is probably mediated by the dopaminergic system as the low affinity for the H_1_ receptor argues against an involvement of this receptor in the inhibitory action on locomotor activity.

In conclusion, our in vivo studies show that 2-bromoterguride is effective in tests for positive symptoms of schizophrenia. In addition, 5-HT_2A_ receptor blockade by 2-bromoterguride has functional relevance in vivo and the induced Fos protein expression in the NAc and mPFC suggests an atypical character. Chronic treatment failed to alter metabolic parameters which are often associated with atypical APDs. 2-Bromoterguride, a drug with partial agonist effects at D_2_ receptors and high affinity for 5-HT_2A_ and α_2C_-adrenergic receptors, is a promising candidate for antipsychotic treatment. Further preclinical behavioral studies predicting antipsychotic effects are needed to verify 2-bromoterguride as an atypical APD, in particular experiments with relevance to cognitive impairment and negative symptoms present in schizophrenia.
